# Zebrafish: A Model Organism for Studying Enteric Nervous System Development and Disease

**DOI:** 10.3389/fcell.2020.629073

**Published:** 2021-01-21

**Authors:** Laura E. Kuil, Rajendra K. Chauhan, William W. Cheng, Robert M. W. Hofstra, Maria M. Alves

**Affiliations:** ^1^Department of Clinical Genetics, Erasmus University Medical Centre, Rotterdam, Netherlands; ^2^Stem Cells and Regenerative Medicine, University College London (UCL) Great Ormond Street Institute of Child Health, London, United Kingdom

**Keywords:** Hirschsprung disease, gut transit, CRISPR/Cas9, morpholino, functional genetics, drugscreen, gastrointestinal system, zebrafish

## Abstract

The Enteric Nervous System (ENS) is a large network of enteric neurons and glia that regulates various processes in the gastrointestinal tract including motility, local blood flow, mucosal transport and secretion. The ENS is derived from stem cells coming from the neural crest that migrate into and along the primitive gut. Defects in ENS establishment cause enteric neuropathies, including Hirschsprung disease (HSCR), which is characterized by an absence of enteric neural crest cells in the distal part of the colon. In this review, we discuss the use of zebrafish as a model organism to study the development of the ENS. The accessibility of the rapidly developing gut in zebrafish embryos and larvae, enables *in vivo* visualization of ENS development, peristalsis and gut transit. These properties make the zebrafish a highly suitable model to bring new insights into ENS development, as well as in HSCR pathogenesis. Zebrafish have already proven fruitful in studying ENS functionality and in the validation of novel HSCR risk genes. With the rapid advancements in gene editing techniques and their unique properties, research using zebrafish as a disease model, will further increase our understanding on the genetics underlying HSCR, as well as possible treatment options for this disease.

## The Mammalian ENS and Its Role in Human Disease

**Figure 1:**
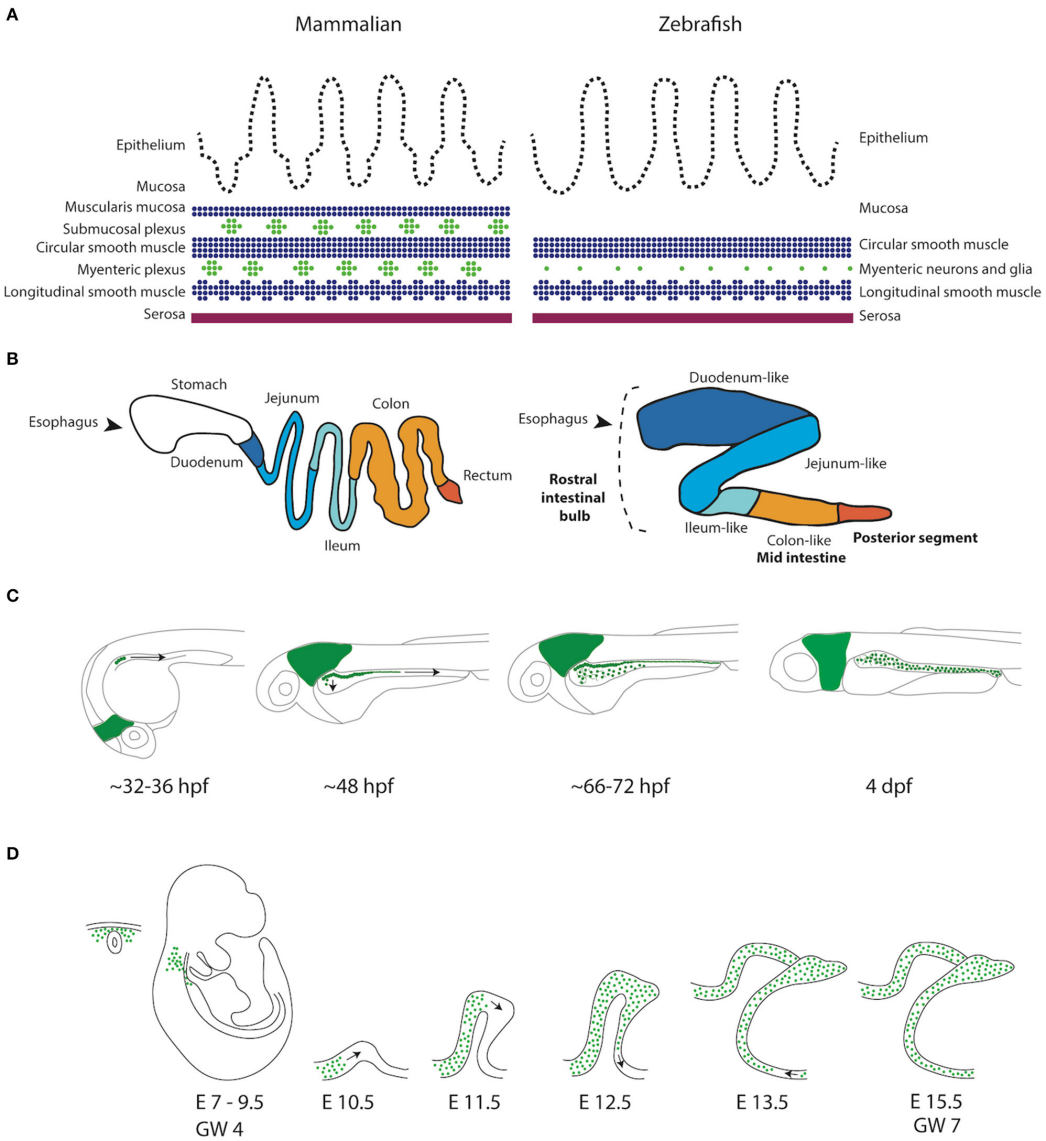
Schematic representation of the GI tract in mammalians and zebrafish. **(A)** Schematic representation of the layers present in the GI tract of mammalians and zebrafish. Zebrafish lack the muscularis mucosa and submucosal plexus. In addition, the enteric neurons are not organized in ganglia, but rather as individual cells (Wallace and Pack, 2003). **(B)** Schematic representation of the mammalian (human) and the zebrafish digestive system. While the human GI tract is divided in stomach, duodenum, jejenum, ileum and colon, the GI tract of the zebrafish is traditionally subdivided in three major components, rostral intestinal bulb, mid intestine and posterior segment (Wang et al., 2010). A new division based on conserved transcriptional profiles has also been proposed, which is depicted by color alterations and labels in regular font (Lickwar et al., 2017). Note that boundaries between sections should not necessarily be considered discrete. **(C)** Schematic representation of ENS development in zebrafish showing that the NCCs depicted in green are first detected in the developing intestine around 32–36 h post-fertilization (hpf). NCCs migrate in two parallel chains caudally, between 36 and 66–72 hpf. Starting rostrally, the NCCs start to migrate laterally to form a network. At 4 days post-fertilization (dpf) the ENS network has been formed around the total length of the intestine. **(D)** Schematic representation of ENS development in mammals. Vagal NCCs colonize the foregut at embryonic day (E)7-E9.5 in mice and gestational week (GW) 4 in humans. From E10.5, NCCs migrate caudally in various multicellular strands.

The gastrointestinal (GI) tract is the core digestive system in our body and has a central role in the absorption of water and nutrients for energy provision. The GI tract is regulated by the enteric nervous system (ENS), which is composed of neurons and glia. In humans, these cells form ganglia along the wall of the GI tract, which are located in the myenteric and submucosal plexuses (Figure 1) (Furness, 2009). The myenteric plexus, also called Auerbach's plexus, lies between the longitudinal and circular muscle layers, and regulates muscle contraction and relaxation needed for peristalsis. The submucosal plexus, or Meissner's plexus, is located between the circular muscle layer and mucosa, and is needed for fluid uptake and secretion, blood flow and homeostasis. The ENS is entirely derived from the neural crest (Furness, 2009). Specifically, neural crest cells (NCCs) migrate from the hindbrain, the vagal region of the neural tube, into and along the entire length of the GI tract (Le Douarin, 1982). A second contribution to the ENS arises from sacral NCCs that seed the distal part of the GI tract with enteric ganglia. Such contribution has been mostly studied in mouse and avian models, but is thought to be present in humans as well (Orts Llorca, 1934; Gershon et al., 1993; Burns and Douarin, 1998; Wallace and Burns, 2005; Wang et al., 2011). Defects in ENS establishment in humans cause a wide range of enteric neuropathies, including Hirschsprung disease (HSCR). HSCR is one of the most common causes of life-threatening intestinal obstruction in neonates. The prevalence of HSCR is 1 in 5.000 newborns, and it affects more males than females (4:1) (Badner et al., 1990). HSCR is a complex genetic disorder for which around two dozen genes have already been identified. It can occur as part of a syndrome, with a mendelian mode of inheritance, but more often in an isolated manner (Amiel et al., 2008). In such cases, HSCR shows complex non-mendelian patterns of inheritance where rare coding variants, pre-disposing haplotypes, copy number variants and, one or more “risk genes” with low penetrance, play a role (Amiel et al., 2008; Alves et al., 2013). HSCR is characterized by the absence of enteric ganglia in the distal part of the GI tract. As a consequence, perturbed peristalsis of the aganglionic colon leads to failure to pass stool. The current pathophysiological model is that failure of NCCs to migrate, differentiate, proliferate or survive, thereby forming a functional ENS network, results in HSCR.

## Zebrafish As a Model Organism for Developmental Disorders

Zebrafish are vertebrates often used for *in vivo* studies, because they are relatively small in size and the embryonic and larval development is *ex utero* and fast, since within 5 days post-fertilization (dpf), all major organ systems are formed (Kimmel et al., 1995). Zebrafish embryos are virtually transparent during development, allowing visualization of internal organs in a non-invasive way. This animal model has also high fecundity, and one breeding pair gives on average, 200 eggs (Lieschke and Currie, 2007). The zebrafish genome has been sequenced, is well annotated and around 71.4% of human proteins have at least, one zebrafish ortholog. This percentage seems to be even higher (82%) for proteins involved in disease development (Howe et al., 2013). Due to recent technical advances, including genome editing, lineage tracing, optogenetics, and *in vivo* imaging, the zebrafish gained popularity as a model for basic research, as well as for disease modeling, in a wide variety of research topics (Lieschke and Currie, 2007). Zebrafish have proven to be a great tool to model human disease, in particular developmental disorders that manifest in the fish within the first 5 days of development. Moreover, large-scale drug- and genetic-screenings can be relatively easily executed in zebrafish, which have a high value for both diagnostic and therapeutic purposes (Horzmann and Freeman, 2018; Paone et al., 2018; Vaz et al., 2018).

## Zebrafish as a Model Organism to Study the ENS

### Conserved Intestinal Organization in Zebrafish

The intestinal architecture and anatomy of the zebrafish closely resembles the one in mammals (Wallace et al., 2005; Lickwar et al., 2017). The intestinal epithelium is folded irregularly into ridges to enlarge the absorptive surface, an organization somewhat reminiscent of the villous epithelium of mammals (Ng et al., 2005; Wang et al., 2010). It is also rich in enterocytes, goblet cells and enteroendocrine cells. However, the zebrafish gut is simpler: it lacks crypts of Lieberkühn, a submucosal layer and myenteric neurons are arranged as neuronal pairs or single neurons, instead of clustered in ganglia (Figure 1) (Wallace et al., 2005; Wang et al., 2010). The zebrafish gut undergoes rapid development and at 5 dpf, the whole GI tract is functional (Wallace and Pack, 2003). As in other vertebrates, the zebrafish ENS is derived from the neural crest (Kelsh and Eisen, 2000). Vagal NCCs migrate as two parallel chains of cells to colonize the whole gut, and differentiate into enteric neurons and glia (Shepherd et al., 2004). There is no evidence to support a sacral NCC contribution to the ENS in zebrafish (Shepherd and Eisen, 2011). However, the zebrafish ENS is comparable to the mouse and human ENS based on gene expression and functional studies (Heanue and Pachnis, 2008; Roy-Carson et al., 2017). By 4 dpf, regular anterograde and retrograde contractions of the intestine occur and can be easily visualized (Holmberg et al., 2003). The enteric innervation is already functional by 5 dpf, when zebrafish larvae start feeding. Major neurotransmitters crucial for gut motility in humans, such as serotonin (5-hydroxytryptamin, 5HT), neurokinin A (NKA), vasoactive intestinal polypeptide (VIP), pituitary adenylate cyclase activating peptide (PACAP), nitric oxide (NO) and calcitonin gene-related peptide (CGRP), are also present in the adult zebrafish gut (Holmberg et al., 2004; Olsson et al., 2008).

The zebrafish transcriptome of the developing ENS, has been determined by bulk RNA sequencing of neural and non-neural crest cells isolated from 7 dpf zebrafish intestines (Roy-Carson et al., 2017). The results obtained showed expression in the zebrafish ENS of known markers of the mammalian ENS, but also revealed previously unidentified genes that are enriched in the ENS. Importantly, this study showed the conserved expression of neuronal genes such as, the ELAV like neuron-specific RNA binding protein 3 (*elavl3*), chimerin 1 (*chn1*) and synapsin 2a (*syn2a*), as well as subtype markers, such as choline acetyltransferase a (*chata*), GABA receptor 1 (*gabbr1*) and vasoactive intestinal peptide (*vip*). Also, the expression of various HSCR-causing genes including the Rearranged during transfection *(ret*), GDNF family receptor alpha 1 (*gfra1)* and zinc finger E-box binding homeobox (*zeb2)*, is conserved (Roy-Carson et al., 2017). More recently, researchers started to determine the neural crest transcriptome at a single-cell resolution in late embryonic, to early larval stage (Howard et al., 2020). They shed light on the transcriptomes of enteric NCCs transforming into enteric neurons, and showed conserved enteric programs (Lasrado et al., 2017; Howard et al., 2020). Their data contain cells in a spectrum of differentiation states, enabling the identification of five subclusters of enteric neurons (Howard et al., 2020). For example, the onset of *elavl3* expression reflects immature neurons, whereas the *nos1*^+^/*vipb*^+^ subpopulation represents cells further along a differentiation trajectory. The zebrafish transcriptome shows overlap with the human and mouse ENS single-cell transcriptome (Drokhlyansky et al., 2020), for example, with regard to the presence of various enteric neuronal subtypes. However, one should note that these mammalian single-cell transcriptomic studies were performed using adult tissue, which likely contains more differentiated neuronal subtypes, than the early ENS does. Nevertheless, these data showed that the zebrafish can offer insights in the earliest specification of enteric neurons and enteric neuronal subtypes, which will further increase our understanding of the ENS development relevant to disease, such as HSCR.

### Visualization of Enteric Neurons

**Figure 2:**
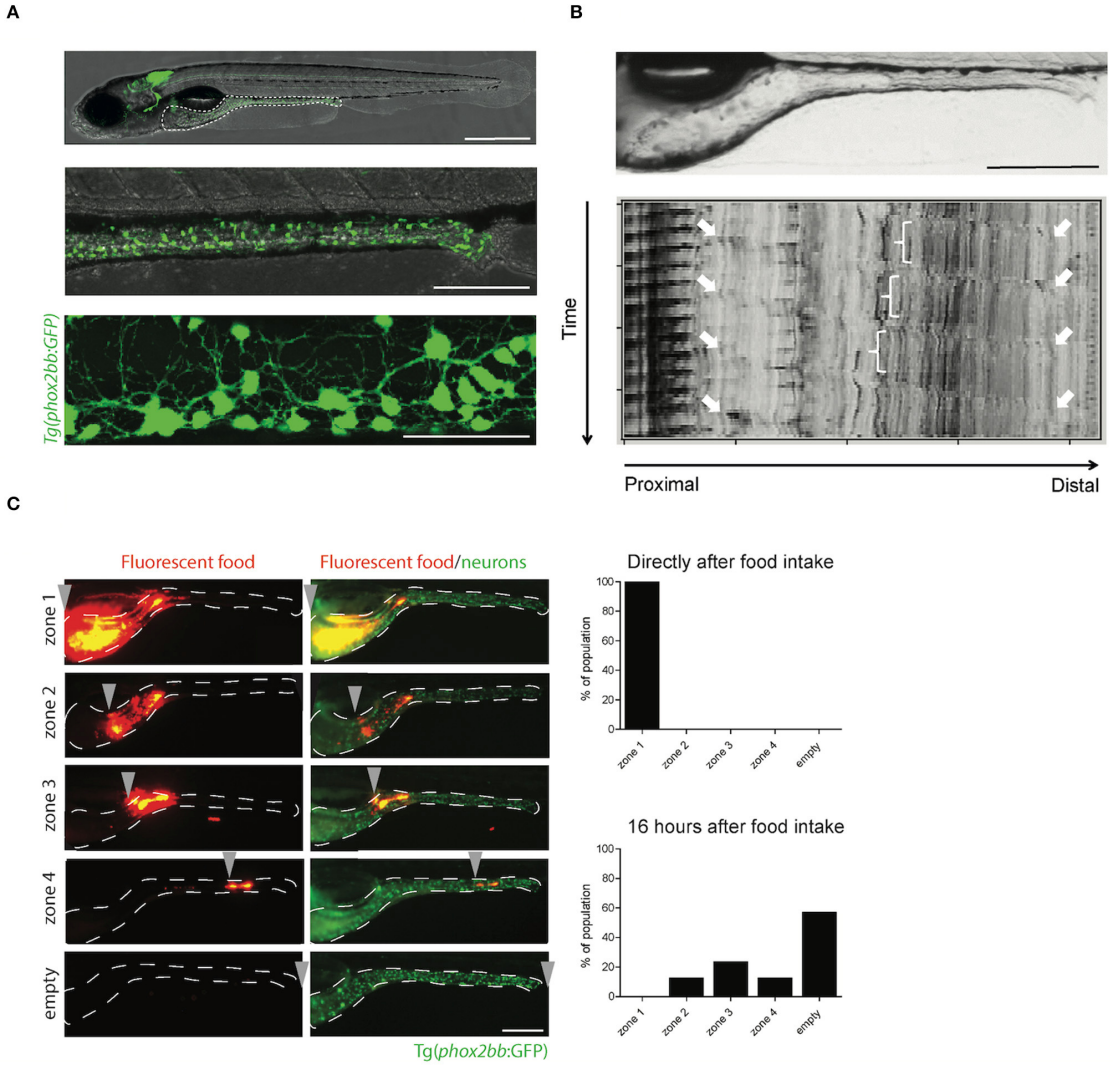
The transparency of zebrafish larvae allows non-invasive visualization of enteric neurons **(A)**, peristalsis **(B)**, and gut transit **(C)**. **(A)** Microscopy images of Tg(*phox2bb*:GFP) zebrafish larvae at 5 dpf. Scale bar represents 500 μm (top), 200 μm (middle) and 50 μm (bottom). **(B)** Still image of the zebrafish intestine (top) and spatiotemporal map (STMap) of gut flow (bottom). Scale bar represents 200 μm. **(C)** Microscopic images of Tg(*phox2bb*:GFP) zebrafish larvae fed with fluorescently labeled food, showing typical intestinal transit and describing the transitional zone. Graphs showing phenotypical scoring of larvae directly after food intake (top graph) and 16 h after food intake (bottom graph). Scale bar represents 200 μm.

Detection of enteric neural crest cells (ENCCs) and enteric neurons in zebrafish, is the vital first step to study ENS development and defects herein. As zebrafish embryos and larvae are transparent, transgenic reporter lines are used to visualize and trace cells in living animals. These reporter lines are widely used in the field and often have a cell-specific promoter or regulatory element, that drives expression of a fluorophore in the cells of interest. The Tg(−*8.3phox2bb*:keade)^em2tg^ and Tgbac(*phox2bb*:eGFP)^w37tg^ reporter lines are frequently used by us and are the most common ones to study the ENS, as they mark all NCCs, including migrating ENCCs and differentiated enteric neurons (Harrison et al., 2014; Boer et al., 2016) (Figure 2A). Alternatively, NCCs and enteric neurons can be labeled using the SAGFF234A gal4 enhancer trap line (Asakawa and Kawakami, 2009), whereas the *sox10* reporter line labels only early progenitors (El-Nachef and Bronner, 2020), and the neural-specific beta tubulin (NBT) promotor labels enteric neurons but not NCCs (Peri and Nusslein-Volhard, 2008; Heanue et al., 2016). Specific neuronal subsets can also be labeled using neurotransmitter reporter lines, for example the *chata* reporter line, in which differentiating enteric neurons are labeled (Nikaido et al., 2018).

Recently, a rather non-conventional way to label enteric neurons was developed by applying a local heat shock to induce a genetic switch of the fluorophore expression, in the zebrabow line (Pan et al., 2013; Kuwata et al., 2019). The shock was applied at the dorsal vagal area of 13-14 hpf embryos, resulting in three different color combinations of enteric neurons (Kuwata et al., 2019). With this method, it was possible to distinguish individual enteric neurons and trace them over extended periods of time. Kaede reporters can also be used to track individual cells by photo-conversion. However, the conversion is not permanent, limiting extended live-imaging experiments.

In general, antibody availability for use in zebrafish is much lower compared to the availability for mammalian samples. Nevertheless, there are a number of antibodies that stain mainly differentiated enteric neurons in larval and adult zebrafish, such as antibodies against Elavl3/HuC/D, choline acetyltransferase (ChAT), serotonin (5-HT), calretinin (CR), calbindin (CB), tyrosine hydroxylase (TH), vasoactive intestinal peptide (VIP), pituitary adenylate cyclase-activating peptide (PACAP) and neuronal nitric oxide synthase (nNOS) (Uyttebroek et al., 2010, 2013). Such antibodies have been used to identify, at least eight neuronal subtypes in zebrafish using combined staining (Uyttebroek et al., 2010, 2013).

### Zebrafish Enteric Glia

Until recently, not much was known about the presence of enteric glia, the non-myelinating glia in the intestine. Enteric glia are involved in intestinal motility and are thought to have the capability to generate new enteric neurons and glia (Joseph et al., 2011; Laranjeira et al., 2011; Grubisic et al., 2018). Zebrafish enteric glia were first identified in the neuropil layer containing glial cells and axons, using transmission electron microscopy (TEM) in larval zebrafish at 7 and 18 dpf (Baker et al., 2019). These enteric glia contained characteristic glial filaments, with either a filamentous appearance or a less filamentous appearance, distinguishing two types of enteric glia. The authors also showed zebrafish Glial fibrillary acidic protein (Gfap) immunohistochemistry staining where, enteric glia processes largely form an inner layer separating neuronal axons from the intestinal epithelium (Baker et al., 2019). However, another report states that the observed Gfap staining likely represents cross-reactivity, as it persisted in *ret* mutant larvae lacking an ENS (McCallum et al., 2020). Moreover, they show that zebrafish enteric glia do not seem to (highly) express the canonical “glial markers” such as, the fatty acid binding protein 7 (Fabp7) and the S100 calcium-binding protein B (s100β). Instead, they are reliably labeled in the Notch activity reporter tg(*her4.3:*EGFP)^y83Tg^ in larval and adult zebrafish. They also show that, zebrafish enteric glia resemble mammalian enteric glia based on their ultrastructural and general morphology, gene expression and location. The *her4.3*+ cells were defined as neural crest derived, post-migratory cells that retain proliferative and neurogenic potential throughout life (McCallum et al., 2020). An independent study, also reported the presence of neurogenic precursors in adult zebrafish, that lack expression of the canonical “glial marker” Gfap (El-Nachef and Bronner, 2020). They performed lineage tracing and found that these enteric glia were not derived from the vagal neural crest, but from the trunk neural crest, more specifically the Schwann cell precursors (El-Nachef and Bronner, 2020). Thus, although zebrafish enteric glia do not express the canonical “glial markers” and Gfap immunohistochemistry might be unspecific for enteric glia in zebrafish, a neurogenic progenitor population with enteric glial characteristics seems to exist in this organism, opening new avenues to study these progenitor cells and explore whether they remain present in HSCR models (El-Nachef and Bronner, 2020; McCallum et al., 2020).

### Gut Motility in Zebrafish Larvae

Gut motility is controlled by the ENS and modulated by different neurotransmitters. Therefore, measuring gut motility can be used to assess the consequences of alterations in ENS development, such as the ones leading to HSCR. Gut motility consists of many features, which include: (1) standing contraction, in which the food is mixed in the stomach; (2) peristaltic movements in anterograde (mouth to anus) and retrograde (vomiting or regurgitation) direction; and (3) phasic contraction of sphincters (Olsson and Holmgren, 2001). In zebrafish, the erratic and spontaneous contraction waves are noticed before the onset of feeding (3 dpf), and later (4–7 dpf) distinct anterograde, retrograde and rectal contractions are observed (Holmberg et al., 2003). Zebrafish lack a stomach, so the retrograde contractions in the anterior intestine may take over the function of food mixing (Figure 1). Retrograde and anterograde contractions spread in both directions from mid intestine, to mainly transport the food along the gut (Holmberg et al., 2003). Gut motility patterns can be imaged in zebrafish larvae *in vivo*, and in a non-invasive manner using real time video microscopy (Figure 2B; Supplementary Video 1). The data obtained can be inferred by spatiotemporal maps (STMaps) of gut flow (Figure 2B) (Holmberg et al., 2007). Such approach has been sucessfully used by us and others, to study gut motility (Holmberg et al., 2004; Kuhlman and Eisen, 2007). A more sophisticated way to analyze not only the temporal frequency of motility, but also displacement along the anterior to posterior, and dorsal to ventral axis, has been recently reported (Ganz et al., 2018). The authors generated a quantitative approach to determine from the STMaps, the frequency and speed of peristaltic waves by generating cross-correlation maps. Thereby, several parameters such as, wave duration, wave speed and amplitude, were able to be measured (Ganz et al., 2018).

### Gut Transit Assay

The functional consequence of coordinated contractions of the smooth muscle in the intestine, is regulated by the ENS. In HSCR patients these contractions are impaired, leading to a failure to pass stool. To measure gut transit, zebrafish larvae are fed with normal larval feed mixed with fluorescent microspheres. These microspheres are non-absorbable, non-digestible and thus tractable, using fluorescent microscopy (Field et al., 2009). Larvae typically exhibit inter-individual differences in the feeding and ingest different amounts of food. Therefore, a pre-sorting is warranted to reduce variability. To regulate food intake in zebrafish, a microgavage method was established, which uses a standard microinjection procedure of microspheres, directly into the lumen of the anterior intestine of larvae (Cocchiaro and Rawls, 2013). This reduces variability due to differences in food intake, but increases the manual labor and expertise required to perform this technique. In addition, the throughput of an experiment will be limited by the number of larvae that can be subjected to microgavage. After ingestion or microgavage of the labeled food, larvae are screened under the fluorescent microscope at different time points, for the presence of fluorescent microspheres in the intestine. Transit time can then be measured by expulsion of the fluorescent beads (Figure 2C). Using this approach, we and others have shown that the degree of enteric neurons loss in zebrafish larvae, correlates with reduced intestinal transit, which is in line with the phenotype presented by HSCR patients (Field et al., 2009; Bernier et al., 2014).

A high throughput set-up to study gut transit time, was recently developed in 7 dpf zebrafish (Cassar et al., 2018). After feeding fluorescently labeled food, single larvae were placed in a 96 wells plate and treated with different compounds. Fecal matter accumulated at the bottom of the plate, was measured over time by a spectrophotometer (Cassar et al., 2018). This method was used to predict GI safety issues of drug candidates, but can also be useful to search for compounds that stimulate bowel movement.

## Genetic Modifications in Zebrafish to Study ENS Development

Large-scale forward genetic screens in zebrafish have led to the identification of new genes and pathways for vertebrate development, including ENS development (Driever et al., 1996; Haffter et al., 1996). One of the earliest zebrafish mutants showing an ENS defect, was identified in a genetic screen for pigmentation defects. This is not surprising since pigment cells are also neural crest-derived. The so-called *cls* (*colorless*) mutant, which serves as a zebrafish model for the Waardenburg-Shah syndrome, lacks pigment cells and shows reduced numbers of enteric neurons, as well as additional neural crest cell defects (Kelsh and Eisen, 2000). In a following study, it was shown that the *cls* locus mapped to *sox10*, a gene required for proper neural crest development that has also been associated to HSCR (Dutton et al., 2001; Sanchez-Mejias et al., 2010). In another study, loss of *sox10* in zebrafish decreased intestinal motility and resulted in an altered microbiome causing inflammation (Rolig et al., 2017). Inflammation, or enterocolitis, is an important aspect of HSCR, accounting for the majority of mortality and morbidity described (Demehri et al., 2013). Interestingly, the authors managed to rescue this phenotype by treating the fish with an anti-inflammatory bacterial strain, or by restoring ENS function by transplanting wildtype NCCs (Rolig et al., 2017). Another ENS zebrafish mutant, *lessen*, showed a reduction of enteric neurons in the distal intestine. The mutation mapped to the mediator complex 24 (*med24)* gene (Pietsch et al., 2006; Shepherd and Eisen, 2011). Later, it was found that the *lessen* mutant displayed delayed onset of intestinal motility and disturbed interstitial cells of Cajal, along with ENS development defects (Uyttebroek et al., 2016). Three other mutants with ENS defects were also found in this forward genetic screen, and are listed in Table 1.

**Table 1:** Overview of the stable zebrafish mutants and morphants presenting an ENS phenotype.

**Name**	**Gene**	**Mutant/morphant**	**Phenotype**
Colorless; SRY-related HMG-box 10 (Kelsh and Eisen, 2000; Dutton et al., 2001)	*sox10*	Mutant	Major loss of enteric neurons and GFP+ glia
Gutwrencher (Kuhlman and Eisen, 2007)	*gws*	Mutant	Reduced numbers of enteric neurons
Gutless wonder (Kuhlman and Eisen, 2007)	*glw*	Mutant	Reduced numbers of enteric neurons, especially in dorsal gut (HSCR)
Lessen (Pietsch et al., 2006; Uyttebroek et al., 2016)	*med24*	Mutant	Reduced numbers of enteric neurons, especially in dorsal gut (HSCR)
Casanova (Dickmeis et al., 2001; Reichenbach et al., 2008)	*sox32*	Mutant	Total lack of enteric neurons
RET proto-oncogene (Heanue et al., 2016)	*ret*	Mutant	Loss of enteric neurons (HSCR)
Sonic you (Reichenbach et al., 2008)	*shh*	Mutant	Lack EPCs in intestine, including anterior part
Mooth muscle-omitted (Reichenbach et al., 2008)	*smo*	Mutant	Lack EPCs in intestine, including anterior part
DNA (cytosine-5)-methyltransferase 1 (Ganz et al., 2019)	*dnmt1*	Mutant	Reduced enteric neurons and disruption of intestinal smooth muscle
Ubiquitin-like, containing PHD and RING finger domains 1 (Ganz et al., 2019)	*uhrf1*	Mutant	Reduced enteric neurons and disruption of intestinal smooth muscle
Glial derived neurotrophic factor (Shepherd et al., 2001)	*gdnf*	Morphant	Major loss of enteric neurons (HSCR)
Paired box 3 (Minchin and Hughes, 2008)	*pax3*	Morphant	Absence of EPCs
Class 3 semaphorin c (Jiang et al., 2015)	*sema3c*	Morphant	Reduced numbers of enteric neurons (HSCR)
Class 3 semaphorin d (Jiang et al., 2015)	*sema3d*	Morphant	Reduced numbers of enteric neurons (HSCR)
IkappaB kinase complex associated protein (Cheng et al., 2015)	*ikbkap*	Morphant	Reduced numbers of enteric neurons (HSCR)
Forkhead box D3 (Stewart et al., 2006)	*foxd3*	Morphant	Fewer EPCs reaching gut, lack of EPCs in gut
ADP-ribosylation factor-like 6 interacting protein 1 (Tu et al., 2012)	*arl6ip1*	Morphant	Reduced numbers of enteric neurons
GDNF-family receptor-α 1 (Shepherd et al., 2004)	*gfra1a;gfra1b*	Morphant	EPCs are formed but do not colonize the gut (HSCR)
Paired-like homeobox 2b (Elworthy et al., 2005)	*phox2bb*	Morphant	Half number of enteric neurons - complete absence in distal part (HSCR)
Indian hedgehog (Sribudiani et al., 2018)	*ihh*	Morphant	Reduced numbers of enteric neurons (HSCR)
Neuregulin 1 (Pu et al., 2017)	*nrg1*	Morphant	Reduced numbers of enteric neurons (HSCR)
Myeloid ecotropic viral integration site 3 (Uribe and Bronner, 2015)	*meis3*	Morphant	EPCs only in anterior gut
Heart and neural crest derivatives expressed 2 (Reichenbach et al., 2008)	*hand2*	Morphant	NCCs are formed, but lack of EPCs in gut
Chromodomain helicase DNA-binding protein 8 (Bernier et al., 2014)	*chd8*	Morphant	Reduced enteric neurons in hindgut (HSCR)
DENN Domain Containing 3 (Gui et al., 2017)	*dennd3*	Morphant	Enteric neurons absent in distal end colon (HSCR)
Nicalin precursor (Gui et al., 2017)	*ncln*	Morphant	Enteric neurons absent in distal end colon (HSCR)
Nucleoporin 98 (Gui et al., 2017)	*nup98*	Morphant	Enteric neurons absent in distal end colon (HSCR)
Thymus, brain, and testis associated (Gui et al., 2017)	*tbata*	Morphant	Enteric neurons absent in distal end colon (HSCR)
Mitogen-activated protein kinase 10 (Heanue et al., 2016)	*mapk10*	Morphant	Reduced numbers of enteric neurons (HSCR)
Double-strand-break repair protein rad21 (Bonora et al., 2015)	*rad21a*	Morphant	Fewer enteric neurons posterior end of the gut (CIPO)
Endothelin Receptor Type B (Tilghman et al., 2019)	*ednrbb*	Morphant	Reduced numbers of enteric neurons (HSCR)

**Figure 3:**
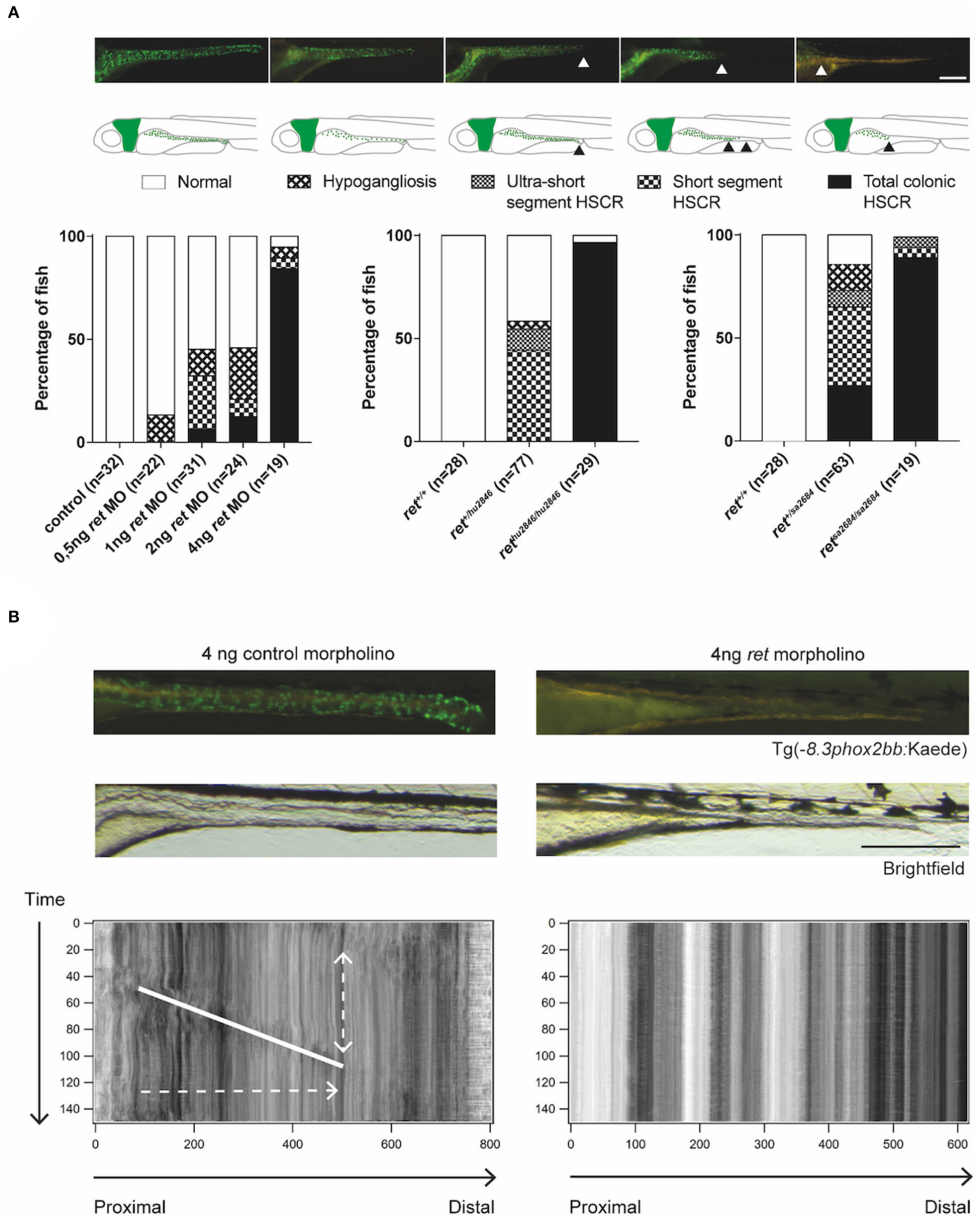
Functional analysis of *ret* morphants and mutants. **(A)** Phenotypical variability observed in zebrafish, using MO targeting Ret expression and two different zebrafish *ret* mutant lines. Proposed phenotyping classifications are shown by microscopy images and illustrations. Scale bar represents 200 μm. **(B)** Microscopic images of Tg(−8.3*phox2bb*:keade) zebrafish injected with control or *ret MO*, show normal ENS colonization in controls and a total colonic HSCR phenotype in the *ret MO* injected fish. Peristaltic function is disrupted in zebrafish lacking enteric neurons, which is shown by the absence of bowel movement in the STMaps. Scale bar represents 200 μm. Note that the use of the terms hypoganglionosis and HSCR in zebrafish despite the lack of ganglia in this organism, refers to the reminiscent phenotype observed in mammals (humans).

The human Rearranged during Transfection *(RET*) gene is the major gene involved in HSCR. Similarly to humans, loss of *ret* in zebrafish causes a HSCR phenotype, in which the severity of the disease correlates with the affected alleles (Tomuschat and Puri, 2015; Heanue et al., 2016). In line with previous reports (Heanue et al., 2016), we observed that homozygous *ret*^hu2846/hu2846^ larvae show only few enteric neurons in the intestinal bulb (most anterior part of the gut). In several heterozygous *ret*^hu2846/+^ larvae, the enteric neurons are restricted to rostral mid-intestine regions, while others remain mildly affected or even unaffected (Figure 3A). The aganglionosis observed in *ret*^hu2846/hu2846^ larvae was found to be the result of defective migration of ENCCs, rather than defective proliferation or increased apoptosis (Heanue et al., 2016). In addition, there is a clear correlation between the presence of enteric neurons and gut motility (Figure 3B; Supplementary Video 1), reminiscent of the intestinal dysfunction observed in HSCR patients (Heanue et al., 2016). We show here, a similar phenotype using the second zebrafish *ret* mutant (*ret*^SA2684^), generated by the TILLING Sanger Institute Zebrafish Mutation Project (https://www.sanger.ac.uk/resources/zebrafish/) (Figure 3A). Similar to the findings discussed earlier in *sox10* mutants, *ret* mutant fish that present with disturbed gut motility show altered intestinal bacterial population dynamics (Wiles et al., 2016). This further supports the notion that the alterations in microbiome are the result of altered gut motility.

In the next sections, we discuss some of the approaches currently used to genetically modify or block, expression of specific ENS genes using zebrafish, in an attempt to provide insights into the genetics and mechanisms underlying HSCR development.

### Morpholino Based Blockage of RNA Translation

A method that has been fruitful in validating HSCR candidate genes that affect ENS development in zebrafish, is the use of morpholinos (MOs). MOs can target translation or splicing of specific genes, abolishing gene expression. However, they should be used with caution, since it is reported that MOs can induce severe side effects or false positive results (Stainier et al., 2017). Several morphants (morpholino-injected zebrafish) have been described showing an ENS phenotype and the majority of them are listed in Table 1. In a study reporting a large family with five HSCR and two constipation cases, variants in four genes: the lipopolysaccharide-responsive and beige-like anchor protein (*LRBA*), *RET*, the RET ligand glial-derived neurotrophic factor (*GDNF*), Indian hedgehog (*IHH*), and a mediator of IHH signaling GLI family zinc finger 3 (*GLI3*), were identified. MO injections in zebrafish also showed that blocking *ihh*, reduced the number of enteric neurons present in the intestine, confirming its previously described role in ENS development shown in mice, and its reminiscence to HSCR (Ramalho-Santos et al., 2000; Sribudiani et al., 2018).

Expression of bone morphogenic protein 2 (BMP2) was found reduced in intestines of HSCR patients (Huang et al., 2019). Zebrafish *bmp2* morphants showed a major defect in ENS development, which was due to reduced proliferation and differentiation of enteric progenitors (Huang et al., 2019). Interestingly, Bmp2 was found to directly regulate expression of the glial-derived neurotrophic factor (Gdnf), a known HSCR gene and ligand of Ret. This result suggests that alterations in BMP2 expression could disturb ENS development, leading to HSCR.

In the study describing the zebrafish *ret*^hu2846^ mutant, the authors showed that heterozygous *ret*^hu2846/+^ larvae could be used to identify susceptibly loci, by injecting a MO in the heterozygous *ret*^hu2846/+^ and wildtype larvae (Heanue et al., 2016). They found that in a heterozygous *ret*^hu2846/+^ background, blocking expression of the mitogen activated protein kinase 10 gene (*mapk10*) with a MO, increased the loss of enteric neurons in the distal part of the gut (Heanue et al., 2016). The authors also generated a *mapk10* mutant using transcription activator-like effector nucleases (TALENs), which is an efficient way to perform targeted genome editing and can be used in zebrafish (Cermak et al., 2011). However, the *mapk10* mutant fish alone did not show any abnormalities in the ENS, showing that only in the ret^hu2846/+^ background, loss of *mapk10* worsens the ENS phenotype (Heanue et al., 2016).

Based on the various HSCR phenotypes observed in the *ret* mutant and morphant zebrafish, we here introduce a scoring system in larvae to facilitate standardization of observed phenotypes in novel HSCR models (Figure 3A). This system is based on the length of the intestine that lacks neurons, reminiscent of HSCR in humans (total colonic, short segment or ultra-short segment HSCR), or in overall reduction in the number of neurons, reminiscent of hypoganglionosis in humans (hypoganglionic) (Figure 3A).

### Targeted Genome Editing

Targeted genome editing can be used to introduce insertions or deletions (indels) at specific target coding sequences, often leading to gene disruption and expression of non-functional truncated proteins. The latest generation of targeted genome editing uses the clustered regularly interspaced palindromic repeats (CRISPR)- associated Cas9 system (Jinek et al., 2012, 2013; Cong et al., 2013; Mali et al., 2013), and has been successfully adopted to disrupt target genes in zebrafish (Hwang et al., 2013; Jao et al., 2013). Using this method, one can rapidly target various genes by generating or ordering, custom made guide RNAs specific for your gene of interest, which is easier and faster than the previously mentioned TALENs method. The first report using CRISPR/Cas9 to study the ENS came when the autism-associated gene Chromodomain Helicase DNA Binding Protein 8 (*chd8*), was knocked out in zebrafish, leading to a reduction in the number of enteric neurons. Mutations in *CHD8* cause an early-onset form of autism, presenting with a facial phenotype, macrocephaly, and gastrointestinal problems. A similar phenotype was obtained in zebrafish by blocking *chd8* RNA translation/splicing, using a MO (Bernier et al., 2014). Interestingly, disruption of zebrafish orthologs of another autism gene, the SH3 and multiple ankyrin repeat domains 3 (*SHANK3)*, also altered gut motility. However, this was not caused by a loss of ENS cells, but rather due to loss of intestinal serotonin-positive enteroendocrine cells (James et al., 2019).

Due to the high efficiency of the CRISPR/Cas9 system, it is feasible to analyze phenotypes directly in the injected generation (F0) and hence, quickly screen for the phenotype of interest. Based on this idea, rapid, high-throughput screening methods using CRISPR/Cas9 in zebrafish, have been developed with low off-target effects (Shah et al., 2015; Varshney et al., 2015; Hoshijima et al., 2019; Kuil et al., 2019). Such approach was successfully used in our lab to screen 14 novel candidate genes for HSCR. By comparing the effect observed in the ENS when using MOs, to the effect observed when using CRISPR-Cas9, four rare exonic *de novo* variants affecting the genes: DENN Domain Containing 3 (*dennd3*), Nucleolin (*ncln*), Nucleoporin 98 (*nup98*), and Thymus, Brain And Testes Associated (*tbata*), were identified to cause HSCR (Gui et al., 2017). This study confirmed that similar phenotypes can be found using either a morpholino-based approach or the CRISPR/Cas9-system. Furthermore, by disrupting zebrafish orthologs of candidate genes present in large copy number losses identified in HSCR patients, we revealed that HSCR phenotypes can be induced upon loss of the genes: Solute Carrier Family 8 Member A1 (*slc8a1*), Mitogen-Activated Protein Kinase 8 (*mapk8*), T-Box Transcription Factor 2 (*tbx2*), and Ubiquitin Recognition Factor In ER Associated Degradation 1 (*ufd1l)*. In this study, we modeled synergy between *ret* and other genes by injecting an ATG-blocking MO against *ret*, to induce a dose dependent, highly specific HSCR phenotype (Figure 3A) (Heanue and Pachnis, 2008; Kuil et al., 2020). In case of reduced Ret expression by MO, disruption of the Glutamine synthetase gene (*gnl1*) also resulted in increased occurrence of HSCR phenotypes. With these studies, we show that direct (F0) screening in zebrafish is particularly useful for HSCR research, since it enables a fast validation of candidate genes identified in patients (Gui et al., 2017; Sribudiani et al., 2018; Kuil et al., 2020).

Epigenetic factors are often involved in cellular and developmental processes. Therefore, it is not surprising that they also play a role in intestine and ENS development. The gene regulatory systems of the zebrafish intestine are conserved in higher vertebrates (Lickwar et al., 2017), except during morphogenesis, where in zebrafish the GI tract develops from individual organ anlagen, whereas in amniotes it develops from a common endodermal tube (Wallace and Pack, 2003; Wang et al., 2010). The transition of zebrafish embryos from yolk dependency, to a free-feeding larva is a very rapid process, which requires extensive transcriptional and epigenetic regulation (San et al., 2018). To delineate these regulatory processes, a previous study has determined the epigenetic regulation of transcription in zebrafish. By generating a homozygous mutant for the Enhancer of zeste homolog 2 gene (*ezh2*) coding for a histone methyltransferase involved in transcription repression, they showed that this enzyme is essential for intestinal maintenance and survival of zebrafish larvae (San et al., 2018). This result suggested a possible role of this gene in the development and function of the intestinal tract in humans. Such role has recently been confirmed by showing that, EZH2 is involved in intestinal immune response and is even associated to inflammatory bowel disease (Zhou et al., 2019). Another study has shown that loss of genes coding for the ubiquitin-like protein containing PHD and RING finger domains 1 (Uhrf1), and DNA methyltransferase 1 (Dnmt1) reduced the number of enteric neurons and disrupted intestinal smooth muscle in zebrafish (Ganz et al., 2019). Although epigenetic mechanisms, including hypomethylation, have been suggested as a disease mechanism for HSCR [reviewed in Torroglosa et al. (2019)], this was the first report of a genetic animal model of aberrant methylation, that showed an effect on ENS development *in vivo* (Ganz et al., 2019). It would be of high interest to investigate the downstream transcriptional consequences of hypomethylation in enteric neurons, to further expand our understanding on HSCR pathogenicity.

## Role of Non-genetic Factors on ENS Development Leading to HSCR

Since genetic factors do not explain all HSCR cases, it is likely that other (non-genetic) factors are involved in HSCR pathogenesis. Due to its large clutch of offspring, small body size, high permeability and rapid development, the zebrafish is an excellent model to screen chemical libraries for therapeutic purposes, or for adverse effects. To identify drugs that could disrupt ENS development, a library of 1,508 compounds was tested in zebrafish embryos (Lake et al., 2013). They discovered that administration of Mycophenolate, an inhibitor of *de novo* guanine nucleotide biosynthesis, led to incomplete colonization of the gut by ENCCs and thus, to impaired ENS development. In another study, zebrafish embryos were used to screen for common medicines frequently taken by women during early pregnancy. It was found that Ibuprofen caused a HSCR-like phenotype, due to reduced migration of NCCs during early development (Schill et al., 2016).

Retinoic acid (RA) plays an important role in collective chain migration of ENCCs and their survival. Therefore, loss of RA leads to a HSCR phenotype caused by stalled migration, followed by detachment and apoptosis (Uribe et al., 2018). Interestingly, this effect was only induced during a “sensitive time period” during development, wherein ENCCs migrate in chains toward the distal end of the gut. On the other hand, supplementing RA stimulated enteric neuron development, leading to increased numbers (Uribe et al., 2018). Since attenuation of RA caused the migratory chain to stall and disorient, RA seems to act as a chemoattractant to guide ENCCs to the distal gut. Vitamin A is the precursor for functional RA and therefore, this process could be affected by altered levels of vitamin A. From several mouse studies it became clear that vitamin A can regulate the number of ENCCs and it is likely that the levels of vitamin A during pregnancy affect the risk of HSCR [reviewed in Heuckeroth (2018)]. Taken together, these studies showed that HSCR phenotypes are not only caused by genetic defects, but also by exposure to certain factors during development such as medication, suggesting that non-genetic mechanisms could affect HSCR occurrence.

## Identification of Drugs That Stimulate ENS Development

Compounds that have a positive effect on ENS formation, i.e., increase the number of enteric neurons, could be valuable to find a potential new therapy for HSCR. Currently, the most promising future therapy for HSCR, seems to be autologous cell transplantation. As mentioned earlier, transplantation of wildtype ENS progenitors in *sox10* zebrafish mutants could rescue the ENS (Rolig et al., 2017). In the *uhrf1* mutant fish, ENS progenitor transplantation from wildtype donors was used to test cell autonomous effects (Ganz et al., 2019). Interestingly, transplanted wildtype cells were absent from the distal-most region in the mutants, showing that Uhrf1 function is necessary in non-neuronal cells in the intestine, for ENS progenitors to migrate to the far end of the intestine (Ganz et al., 2019). In mammalian HSCR models a lot of progress has been made in optimizing transplantation therapy. However, there are still several limitations to overcome such as, limited migration of transplanted cells along the gut, requirement of very high numbers of cells and the integration in the endogenous ENS network [reviewed in: Obermayr and Seitz (2018)]. Combining transplantation with the addition of chemical compounds that stimulate ENS development or make the ENS environment more favorable, could significantly improve transplantation outcomes (Lui et al., 2018; Zhao et al., 2020). The general idea for transplantation in patients, which is currently the major focus of many groups worldwide, is to obtain enteric neuronal progenitor cells and, after *in vitro* expansion, transplant them back to the aganglionic distal colon of the patient. As mentioned in the previous section, retinoic acid increases enteric neuron numbers *in vivo* and is currently also used *in vitro* to enhance specification of enteric neural progenitors (Frith et al., 2020). During a chemical screening in cultured human ENCCs, pepstatin A was discovered to be capable of improving colonization of transplanted NCCs *in vitro* and *in vivo* (Fattahi et al., 2016). Similarly, we demonstrated increased NCC survival and growth in a chemically induced HSCR rat model, by using a ROCK inhibitor (Zhao et al., 2020). Another study found that administration of glial cell derived neurotrophic factor (GDNF) rescued megacolon, prevented death and stimulated ENS regeneration in the *Holstein* (a model for trisomy 21-associated HSCR), *TashT* (a model for male-biased HSCR) and *Piebald-lethal* (a model for *EDNRB* mutation- associated HSCR) mice models (Soret et al., 2020). These results suggest that factors expressed by cells other than enteric neurons, are able to promote proliferation and engraftment of ENCCs. In addition, they prompt us with candidate compounds to use for improvement of future transplantation therapy.

To date, there is no chemical screen performed in zebrafish described to identify compounds that could prevent, or even revert the HSCR phenotype. However, mutant zebrafish lines that exhibit HSCR or other ENS phenotypes, are available and could be used for this purpose. Such study would be highly informative, as the complex ENS developmental process would be studied *in vivo*, as a whole, in developing zebrafish. This could result in the identification of chemicals that could facilitate ENS development, by targeting not only neuronal progenitors and neurons, but also other cell types, in the developing gut.

## Conclusions

Despite many years of research on ENS development, we still lack a complete understanding of all the genes involved and causative of HSCR. Human genetic studies using Next Generation Sequencing approaches have revealed many new HSCR candidate genes. However, to better understand the pathogenicity of variants in these novel genes, and how they influence ENS development, a fast and robust method to functionally validate their effect, is in demand. The zebrafish is an excellent model to study the ENS and subsequently, enteric neuropathies such as HSCR, as rapid transgenic techniques, high-resolution fluorescent *in vivo* imaging, and well-characterized promoters for tissue-specific expression, are already available. Gene expression can also be easily disrupted or modified in zebrafish, to study the effect of specific genes, or a combination of genes, on the ENS by evaluating neuronal count, gut motility and intestinal transit time. Furthermore, zebrafish provides a high-throughput system for targeted validation and potential treatment strategies, using genome-editing technologies and drug screening set-ups. As mentioned in this review, the current general belief is that a defect in either migration, differentiation, proliferation or survival of enteric progenitors, causes the developmental defects observed in HSCR patients. Zebrafish studies have been instrumental in increasing our knowledge on ENS development and how genetic defects affect these processes, leading to HSCR.

## Future Perspectives

The advances in using CRISPR/Cas9 for genome editing allowed for a rapid expansion of the genetic toolbox available to edit the genome. The efficient and relatively easy generation of zebrafish mutants using these techniques, enhances tremendously the potential of this animal model for disease modeling. In combination with a variety of (functional) readouts, it is easy and more importantly, rapid to screen for ENS related phenotypes.

Up until now, most studies validate potential human disease candidate genes by introducing frameshift mutations, which will most of the time disrupt protein function. However, when patients have a point mutation that does not lead to a truncated protein, or might even lead to a gain of function, one needs another method to study this effect. In case the amino acid sequence containing the mutation is conserved in zebrafish, several approaches can be used to make a point mutation, leading to the same amino acid change observed in the patient. For example, a regular Cas9 protein can be injected together with a specific guide RNA targeting the position of the point mutation, and a single-stranded oligo or plasmid containing the mutation of interest and specific homology arms [Reviewed in: Albadri et al. (2017)]. However, since homologous directed repair (HDR) does not occur frequently in zebrafish, non-homologous end joining seems to be favored over HDR, as a repair mechanism. This leads to a relatively low efficiency of correction of specific point mutations, lacking unwanted indels, or insertion of the complete oligo with homology arms. Recent progress in CRISPR-mediated gene editing, currently allows the introduction of point mutations using base editors, cytidine deaminases, which are attached to a modified Cas9 protein. This technique can make the conversion of G-C base pairs to A-T base pairs or vice-versa, without generating double strand breaks [reviewed in: Liu et al. (2019)]. In zebrafish, sequence-specific single base mutations were generated with an efficiency ranging from ~9 to 28%, and a low number of indels (Zhang et al., 2017). The possibility of targeted knock-in single nucleotides in zebrafish, is valuable to analyze specific variants identified in HSCR patients, especially in the case of missense variants, when the CRISPR/Cas9 knockout might not accurately reflect the functional consequences of such variants. In addition, in cases where it is impossible to predict the effect of specific amino acid changes, the zebrafish can be a powerful and cost-effective model to study patient specific variants *in vivo*.

Although a lot of effort has been made to study ENS development in zebrafish, some developmental dynamics remain unsolved. For example, with respect to ENS lineages and differentiation paths of enteric glia, enteric progenitors and enteric neurons. In addition, various open questions in the field persist such as: do enteric neuronal stem cells exist in adults? Do new enteric neurons develop from only one, or a few enteric neuronal precursor cells at the wave front, or do they expand in parallel? CRISPR-Cas9 is currently used as scars or barcodes in the genome to reconstruct cell lineages during development, in an organism-wide manner using several techniques (McKenna et al., 2016; Alemany et al., 2018; Spanjaard et al., 2018). To date, these techniques have not been used to trace NCCs or other components of the ENS in particular. However, by doing so, one would be able to answer various open questions in the field, such as those mentioned earlier.

Taken together, the work discussed here shows that the zebrafish is an excellent model to study HSCR, but also for human pediatric research, as it will provide cues to improve our understanding of the complex processes involved in human development. We also believe that the zebrafish will be fruitful in the development of new approaches to treat HSCR. With novel technologies being regularly developed, this animal model promises exciting research opportunities in the future.

## Author Contributions

LEK, RKC, RMWH, and MMA conceptualized the study. LEK, RKC, and WWC wrote the manuscript. All authors read and revised the manuscript and approved the submitted version.

## Conflict of Interest

The authors declare that the research was conducted in the absence of any commercial or financial relationships that could be construed as a potential conflict of interest.
